# Ga-induced electron pump optimization of Fe active sites in NiFe-LDHs for efficient alkaline water electrooxidation

**DOI:** 10.1039/d6sc00682e

**Published:** 2026-06-18

**Authors:** Yu Shuai, Shucheng Liu, GangBiao Li, Yi Liu

**Affiliations:** a School of Physical Sciences, Guizhou University Guiyang 550025 China yliu9@gzu.edu.cn

## Abstract

The development of efficient and stable electrocatalysts for the alkaline oxygen evolution reaction (OER) is crucial for large-scale industrial application in producing green hydrogen. Herein, we propose a targeted electronic modulation strategy to enhance the OER performance of NiFe layered double hydroxides (LDHs) *via* Ga^3+^ doping. Preliminary theoretical analyses indicate that Ga^3+^, serving as a strong Lewis acid center with high electron-withdrawing ability, effectively optimizes the electronic structure of adjacent Fe active sites through indirect orbital interactions. As a result, the synthesized NiFeGa-LDH anode exhibits a low overpotential of only 275 mV at 50 mA cm^−2^ in 1 M KOH and, notably, exhibits stable operation for over 100 hours at an industrial-level current density of 1 A cm^−2^ in an anion exchange membrane (AEM) electrolyzer test (configured as NiFeGa-LDHs//Pt-C), demonstrating overall performance superior to that of the commercial RuO_2_//Pt-C benchmark. Through a combination of theoretical and experimental characterization, we demonstrate that Ga^3+^ acts as an “electron pump” to effectively tune and stabilize the Fe active sites. *Operando* spectroscopic analyses further confirm that this electronic modulation drives an earlier and more extensive electrochemical reconstruction of the catalyst into the active γ-NiFeOOH phase, which is identified as the key structural origin of the activity enhancement. This work pioneers a targeted electronic structure engineering tactic *via* strong Lewis acid doping, providing a transformative pathway for designing advanced electrocatalysts beyond the OER.

## Abstract 2

A targeted regulation strategy utilizing the pronounced electron-withdrawing capacity of Ga^3+^ as a Lewis acid to tune the electronic structure of Fe sites, thereby boosting the intrinsic OER activity.

## Graphical abstract

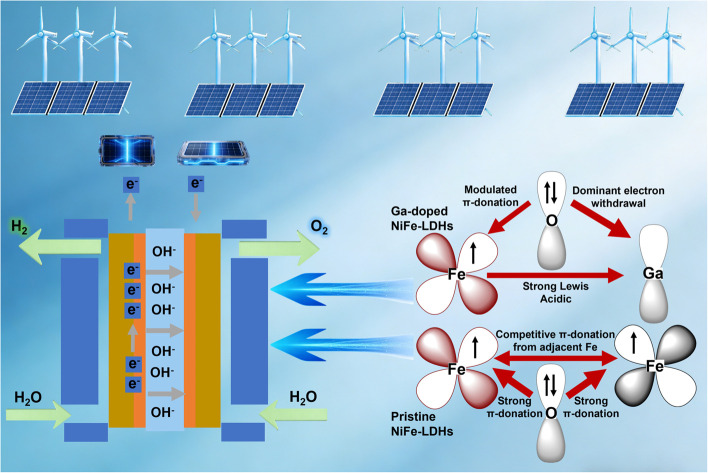

## Introduction

Hydrogen is a promising clean energy carrier for transitioning to a greener and more sustainable energy system. Among various production pathways, water electrolysis powered by renewable electricity – yielding “green hydrogen” – is a key route for large-scale clean hydrogen production.^1,2^ However, the anodic oxygen evolution reaction (OER), a sluggish four-electron transfer process, suffers from slow kinetics,^3–5^ which severely limits the overall energy efficiency of water electrolysis and hinders its industrial-scale deployment. Therefore, developing efficient and stable OER catalysts is crucial for reducing the energy consumption of electrolysis and advancing the green hydrogen economy.

Among numerous non-precious metal catalysts, nickel-iron layered double hydroxides (NiFe-LDHs) are highly promising practical candidates due to their tunable composition and structure, as well as their excellent intrinsic OER activity in alkaline media, which rivals that of precious metals.^6–10^ Substantial research efforts to enhance their performance have long followed two complementary strategies: structural engineering to maximize active site exposure (*e.g.*, *via* exfoliation,^11^ defect creation,^12^ or interlayer expansion^13^) and electronic structure modulation to improve the intrinsic activity of the catalytic sites themselves. A common approach for electronic modulation involves introducing heteroatom dopants (*e.g.*, Cr, Mo, Mn, and V),^14–17^ which are often used to tune the electronic structure of adjacent Ni/Fe sites and thereby regulate the OER energy barrier. However, many of these dopants possess ionic radii or chemical properties distinct from those of the host cations, which can introduce significant lattice strain or non-specific defects.^14,15^ This complication not only obscures the precise attribution of activity enhancements but, more fundamentally, also precludes a clean targeted electronic modulation of the catalytic sites, which are often the performance-limiting factor. This fundamental limitation leaves the full catalytic potential of NiFe-LDHs, especially the contribution governed by the Fe sites, largely untapped, thereby creating a pressing need for a new design strategy. Conventional structural modifications or broad compositional tuning often fail to address the strong binding of oxygenated intermediates on Fe centres, a key factor that hinders desorption and thus limits OER kinetics.^18–22^ Moreover, while previous studies have predominantly focused on modifying Ni sites,^23–27^ the targeted and precise electronic modulation of Fe sites remains a formidable challenge.

To address this challenge, we propose a Ga^3+^-doping strategy for the targeted electronic modulation of the critical Fe sites within NiFe-LDHs while synergistically optimizing the catalytic interface. Our approach is underpinned by a synergistic design principle. First, we capitalize on the physicochemical compatibility—similar ionic radius and precipitation pH—between Ga^3+^ and Fe^3+^ to achieve lattice-matched substitution specifically at Fe sites. Second, leveraging its nature as a strong Lewis acid center, Ga^3+^ is expected to modulate the electronic structure of adjacent Fe sites *via* indirect orbital interactions, thereby optimizing the adsorption strength of reaction intermediates. This targeted perturbation is anticipated to not only optimize the Fe centers but also induce a beneficial electronic reorganization across the local Ni–O structure, thereby collectively tuning the adsorption strength of reaction intermediates. Our preliminary theoretical analyses—including Bader charge, charge difference, and crystal orbital Hamiltonian population (COHP) calculations—reveal that the strongly Lewis-acidic Ga^3+^ forms a highly covalent Ga–O bond. Through the bridging oxygen, this interaction selectively weakens the Fe–O bond at the active site and attenuates the π-donation from oxygen to Fe, thereby favoring the formation of high-valence Fe species, which are considered essential for OER activity. As a result, we synthesized Ga-doped NiFe-LDHs *via* a solvothermal method. The NiFeGa-LDH anode achieved a low overpotential of only 275 mV at 50 mA cm^−2^ (1 M KOH) and, more critically, exhibited stable operation for over 100 hours at an industrial-level current density of 1 A cm^−2^ in anion exchange membrane (AEM) electrolyzer tests (configured as NiFeGa-LDHs//Pt-C), demonstrating overall performance superior to that of the commercial RuO_2_//Pt-C benchmark. Subsequent experimental characterization corroborates the theoretical model by consistently indicating an elevated oxidation state and a modulated electronic structure of Fe upon Ga doping, accompanied by a reduced magnetic moment—all aligning with the predicted electronic redistribution. Furthermore, Gibbs free energy profiles from DFT calculations demonstrate that the optimized electronic structure lowers the energy barrier of the potential-determining step by stabilizing key oxygen-containing intermediates. *Operando* spectroscopic analyses further corroborate that this electronic modulation facilitates a more facile electrochemical reconstruction of the catalyst surface into the active γ-NiFeOOH phase under operating conditions. This modulated electronic environment thereby accelerates the reaction kinetics, providing a fundamental explanation for the enhanced activity and stability.

## Results and discussion

**Fig. 1:**
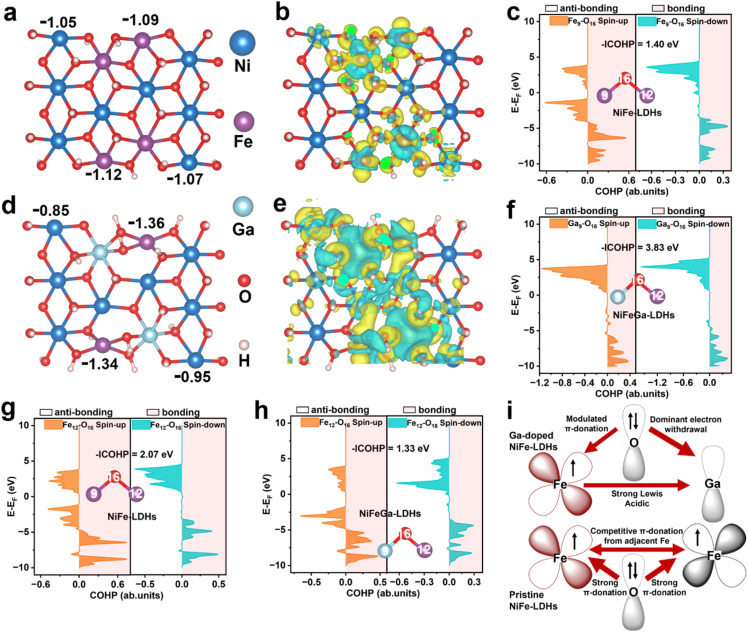
The atomic structure and Bader charge analysis of (a) NiFe-LDHs and (d) NiFeGa-LDHs. Charge density difference of (b) NiFe-LDHs and (e) NiFeGa-LDHs. Comparative crystal orbital Hamilton population (COHP) analysis near the active Fe site in (c and g) NiFe-LDHs and (f and h) NiFeGa-LDHs. (i) Schematic illustration of the electronic structure modulation *via* Ga^3+^ doping in NiFe-LDHs.

To investigate the preferred doping site of Ga^3+^, we constructed supercells with the same chemical composition (Ni : Fe : Ga ratio) but different periodic substitution configurations, as shown in Fig. S1a–c. Among these, the configuration where Ga occupies the specific Fe site depicted in Fig. S1c yields the lowest formation energy (−320.59 eV, Fig. S1d), which is obviously lower than those of alternative configurations (*e.g.*, −319.25 eV and −318.87 eV). This unequivocally demonstrates that, among the possible Fe substitution sites, the Ga^3+^ ion preferentially occupies the specific local environment depicted in Fig. S1c. Therefore, all subsequent electronic structure analyses are based on this thermodynamically preferred model, ensuring the physical relevance of the identified mechanism. Building on this stable structural foundation, we performed density functional theory (DFT) calculations on the optimized pristine and Ga-doped NiFe-LDH models (Fig. 1). Bader charge analysis reveals that Ga doping induces a pronounced electron redistribution: the Fe site loses electron density (from −1.09 to −1.36 e), while the Ni site gains electron density (from −1.05 to −0.85 e), suggesting an elevated oxidation state for Fe and a reduced state for Ni (Fig. 1a and d). The differential charge density plots further visualize this enhanced charge transfer among Ni, Fe, and Ga atoms, with yellow and cyan regions representing electron accumulation and depletion, respectively (Fig. 1b and e).

To decipher the nature of this electronic reconstruction, we performed crystal orbital Hamiltonian population (COHP) analysis around the dopant. In marked contrast to the weaker Fe_9_–O_16_ interaction (–ICOHP = 1.40 eV) in the undoped structure (Fig. 1c), Ga doping introduces a highly covalent Ga_9_–O_16_ bond, evidenced by a markedly elevated –ICOHP value of 3.83 eV (Fig. 1f). This pronounced enhancement underscores the distinct bonding character induced by the strong Lewis acid center. Consequently, the Fe_12_–O_16_ bond at the adjacent active site exhibits a significant reduction in covalent character, as reflected by the decrease in its –ICOHP from 2.07 eV in the pristine system to 1.33 eV upon Ga doping (Fig. 1g and h). Consistently, a similar Ga–O–Ni configuration shows an equally strong Ga–O bond and a corresponding weakening of the adjacent Ni–O bond (see SI Fig. S2). Collectively, these results reveal a coherent mechanism: the strong Lewis acid Ga^3+^, by forming highly covalent Ga–O bonds, withdraws electron density from the bridging oxygen lattice. This specific Ga–O–Fe bridging configuration not only directly attenuates the π-donation from oxygen to Fe—thereby stabilizing the crucial high-valence Fe species^28,29^ (*e.g.*, Fe^4+^, Fig. 1i)—but also concomitantly induces a charge-compensating reduction of the neighboring Ni sites. This synergistic electronic modulation of both active metal centers is a key factor contributing to the enhanced OER activity.

**Fig. 2:**
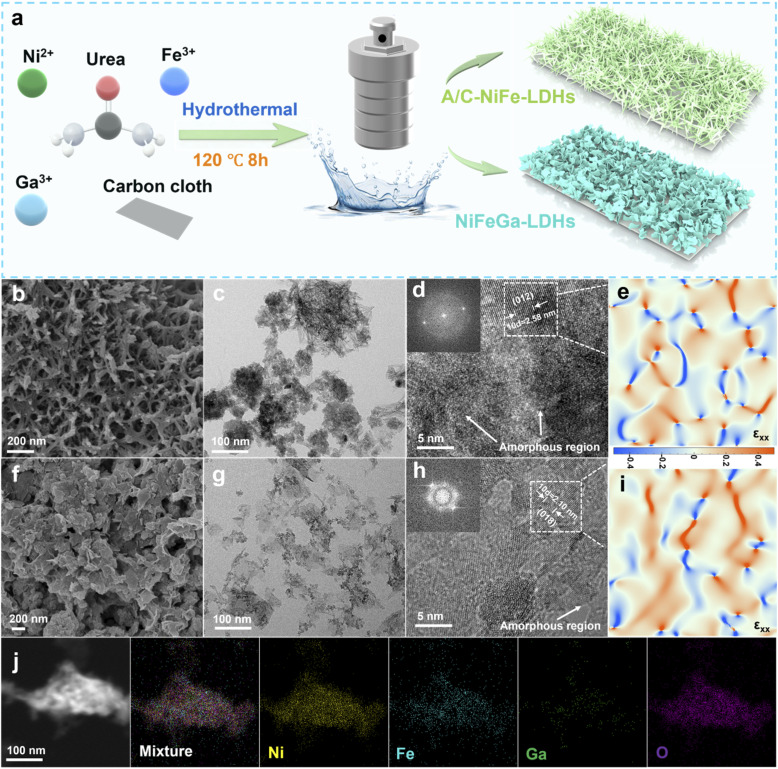
(a) Schematic diagram of the catalyst synthesis. (b–e) A/C-NiFe-LDHs: (b) SEM image, (c) TEM image, (d) HRTEM image with the corresponding fast Fourier transform pattern (inset), and (e) *ε*_*xx*_ geometric phase analysis (GPA) mapping derived from the dashed area in (d). (f–i) NiFeGa-LDHs: (f) SEM image, (g) TEM image, (h) HRTEM image with the FFT pattern (inset), and (i) *ε*_*xx*_ GPA mapping from the dashed area in (h). (j) TEM and elemental mapping of NiFeGa-LDHs.

Based on the mechanistic insights from DFT calculations, we synthesized NiFeGa-LDHs on carbon cloth *via* a solvothermal method, with an undoped NiFe-LDH sample prepared in parallel for direct comparison (Fig. 2a). Remarkably, the scanning electron microscopy (SEM) images show a fundamental morphological transformation upon Ga doping, from a three-dimensional coral-like network of interwoven branches to an assembly of well-defined two-dimensional nanosheets (Fig. 2b and f), indicative of an altered nucleation and growth pathway that yields a more planar and stacked microstructure. Notably, at the micron-scale resolution presented here, no significant difference in overall particle aggregation or macrostructural morphology is evident between the two samples (Fig. S3), indicating that the doping-induced morphological reorganization primarily occurs at the nanoscale. Transmission electron microscopy (TEM) analysis further elucidates these nanoscale differences. While the undoped sample exhibits a dark densely agglomerated particulate morphology, indicative of a growth process favoring compact 3D aggregates (Fig. 2c), the Ga-doped sample displays a lighter more dispersed flake-like architecture (Fig. 2g). This provides direct evidence for the transition to a 2D growth mode, yielding thinner and more separated nanosheets upon Ga doping. High-resolution transmission electron microscopy (HRTEM) analysis provides direct evidence for the crystallographic evolution induced by Ga doping. For the undoped A/C-NiFe-LDHs (where A/C denotes an amorphous/crystalline mixed phase) (Fig. 2d), the image shows a coexistence of limited crystalline domains and predominant amorphous regions. Within the crystalline areas, lattice fringes with a spacing of 2.58 Å, corresponding to the (012) planes, are discernible. The corresponding FFT pattern (inset) exhibits diffuse diffraction rings, characteristic of nanocrystallites embedded in a disordered matrix. In stark contrast, the NiFeGa-LDH sample (Fig. 2h) displays a markedly higher density of lattice fringes and a significantly reduced amorphous background. The measured interplanar spacing of 2.10 Å is indexed to the (018) planes. Notably, the FFT pattern (inset) now shows sharper diffraction spots, clearly indicating moderately improved crystallinity after Ga doping. These observations collectively imply that the incorporation of Ga^3+^ ions promotes structural ordering, facilitating the formation of a more crystalline LDH phase. Geometric phase analysis (GPA) was conducted on selected crystalline regions to assess crystal development and lattice distortion. The geometric phase analysis (GPA) map of the A/C-NiFe-LDHs (Fig. 2e, S4a and b) reveals a complex and heterogeneous strain distribution within the crystalline regions, as reflected by the wide variation in color. This non-uniform strain field indicates the presence of significant localized lattice distortions and incoherent crystal development. In comparison, the GPA map of the Ga-doped sample (Fig. 2i, S4c and d) shows a discernibly more uniform color distribution, indicating a comparatively more homogeneous lattice-strain state. This observed trend toward strain homogenization aligns with and supplements the HRTEM observations, collectively suggesting that Ga doping fosters more coherent crystal development and helps alleviate local lattice distortions. The resulting more ordered and uniformly strained structure is anticipated to favorably influence the local electronic environment and, consequently, the catalytic activity.^30,31^ Finally, energy-dispersive X-ray spectroscopy (EDS) elemental mapping (Fig. 2j) confirms the homogeneous incorporation of Ga within the nanosheet architecture. The spatially coincident signals of Ni, Fe, O, and Ga demonstrate that Ga is atomically dispersed in the LDH matrix without phase segregation.

**Fig. 3:**
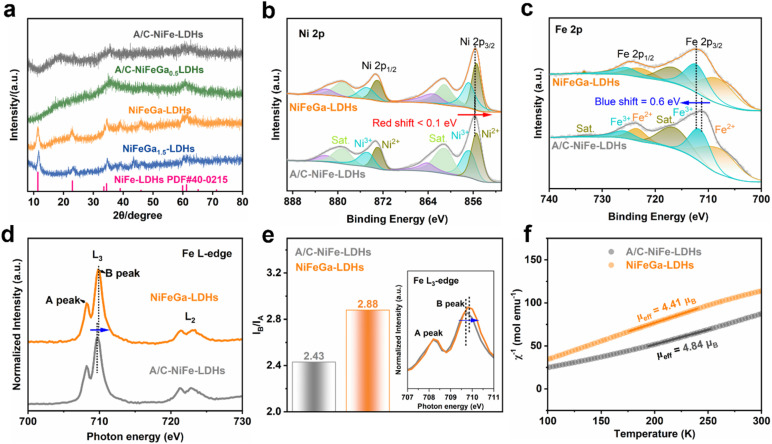
(a) X-ray diffraction (XRD) patterns of different samples; (b) Ni 2p and (c) Fe 2p XPS spectra of A/C-NiFe-LDHs and NiFeGa-LDHs. (d) Fe L-edge XANES spectra of A/C-NiFe-LDHs and NiFeGa-LDHs. (e) Fe L_3_-edge XAS spectra and the corresponding *I*_B_/*I*_A_ peak intensity ratio for A/C-NiFe-LDHs and NiFeGa-LDHs. (f) Temperature-dependent magnetic susceptibility of A/C-NiFe-LDHs and NiFeGa-LDHs.

X-ray diffraction (XRD) analysis was employed to examine the structural evolution upon Ga doping. As shown in Fig. 3a, all synthesized samples exhibit the characteristic diffraction patterns of the layered double hydroxide (LDH) phase. The diffraction peaks observed at 11.41°, 22.97°, 33.54°, 34.43°, 38.99°, 45.99°, 59.94°, and 61.25° can be indexed to the (003), (006), (101), (012), (015), (018), (110), and (113) crystal planes of orthorhombic NiFe-LDHs (PDF#40-0215), respectively. The undoped A/C-NiFe-LDHs display broadened peaks with relatively low intensity, indicative of a nanocrystalline or partially disordered structure. Notably, with increasing Ga content, these diffraction peaks gradually become sharper and more intense, particularly for the NiFeGa-LDH and NiFeGa_1_._5_-LDH samples. This trend clearly demonstrates that Ga incorporation enhances the crystallinity and long-range structural order of the LDHs. Furthermore, a slight but consistent shift in the peak positions is observed, confirming the successful integration of Ga^3+^ ions into the host lattice. No impurity phases are detected, attesting to the phase purity and the formation of a homogeneous solid solution. The surface electronic structure and chemical states were probed by X-ray photoelectron spectroscopy (XPS). Full XPS spectra and Ga 2p and C 1s spectra of NiFe-LDHs and NiFeGa-LDHs are presented in Fig. S5a–c. The high-resolution Ni 2p spectra (Fig. 3b) show that both A/C-NiFe-LDHs and Ga-NiFe-LDHs exhibit two characteristic peaks at 855.6 eV and 873.4 eV, corresponding to Ni 2p_3/2_ and Ni 2p_1/2_, respectively.^14^ Compared to A/C-NiFe-LDHs, the peaks for Ga-NiFe-LDHs shift slightly towards lower binding energy, suggesting a moderate electron enrichment around Ni sites induced by Ga incorporation. In stark contrast, the main Fe 2p_3/2_ and Fe 2p_1/2_ peaks, located at ∼711.3 eV and ∼723.9 eV, respectively, show a pronounced positive shift of approximately 0.6 eV toward higher binding energy for the Ga-doped sample (Fig. 3c).^32^ This distinct shift indicates a depletion of electron density at Fe sites, attributed to the strong Lewis acidity of the incorporated Ga^3+^ ions, which exerts an electron-withdrawing effect, promoting charge transfer from Fe to neighboring Ga and effectively increasing the oxidation state of Fe. Deconvolution of the O 1s spectra (Fig. S5d) reveals four components: lattice oxygen (O_L_, ∼529.6 eV), metal–hydroxyl bonds (O_OH_, ∼531.2 eV), oxygen vacancies (O_V_, ∼532.7 eV), and adsorbed water/hydroxyls (O_H_, ∼533.5 eV). The XPS results collectively indicate that Ga doping induces a significant redistribution of electron density among the metal centers, creating a modulated electronic environment conducive to catalytic processes.^24,32^

To gain deeper insight into the local electronic structure of Fe, soft X-ray absorption spectroscopy (sXAS) was performed at the Fe L_3_,_2_-edge. The Fe L_3_,_2_-edge XAS spectra for the undoped A/C-NiFe-LDHs and Ga-doped NiFeGa-LDHs (Fig. 3d) display the characteristic doublet structure resulting from spin–orbit splitting, characterized by an L_3_ edge centered at approximately 710 eV. The L_3_-edge region shows two primary features (labeled A, ∼708.2 eV and B, ∼709.8 eV) corresponding to transitions from the Fe 2p_3/2_ core level to the crystal-field-split 3d orbitals (t_2g_ and e_g_, respectively) in an octahedral coordination.^33,34^ It is noteworthy that, across all spectra, the intensity of peak B consistently surpasses that of peak A, a spectral signature that is characteristic of Fe^3+^.^35^ Upon Ga doping, a clear shift of the Fe L_3_-edge toward higher photon energy is observed, accompanied by a marked change in the intensity ratio of the e_g_ (B) to t_2g_ (A) features (*I*_B_/*I*_A_) (Fig. 3e). The increase in this ratio reflects a higher relative number of holes in the e_g_ orbitals compared to the t_2g_ orbitals. This preferential electron depletion arises because the e_g_ orbitals are oriented directly toward the coordinating anions, making them more sensitive to changes in ligand-field strength and local oxidation state in high-spin Fe^3+^.^36^ The spectroscopic changes indicate that Ga doping introduces a significant electronic perturbation, facilitating electron depletion from the Fe sites and effectively modulating the local electronic structure of Fe, which harmonizes with the XPS findings. Magnetization measurements provide complementary evidence for the electronic modification. The room-temperature magnetization curves (Fig. S6) for both A/C-NiFe-LDHs and NiFeGa-LDHs exhibit linear reversible paramagnetic-like behavior with negligible hysteresis. A consistent reduction in the magnetization magnitude is observed for the Ga-doped sample, attributable to the dilution of magnetic moments by the incorporation of non-magnetic Ga^3+^ ions. Fig. 3f shows the temperature-dependent magnetic susceptibility (*χ* – *T*) curves under a 1000 Oe field during field cooling (FC). Quantitative analysis *via* the Curie–Weiss law (*χ* = *C*/(*T* − *Θ*)),^37,38^ yielded the parameters *C* = 2.93 emu K and *Θ* = −132 K for A/C-NiFe-LDHs and *C* = 2.43 emu K and *Θ* = −158 K for the NiFeGa-LDH sample. Calculation of the effective magnetic moment (*µ*_eff_) per magnetic ion was performed utilizing the Curie constant (*C*) in accordance with the equation below:1
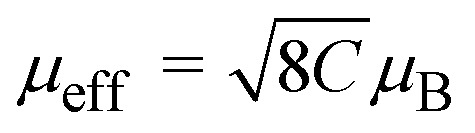


These correspond to average effective magnetic moments (*µ*_eff_) of 4.84 *µ*_B_ and 4.41 *µ*_B_ per magnetic ion, respectively. The significant decrease in the Curie constant (*C*) and the derived *µ*_eff_ upon Ga doping provides complementary evidence for the electronic structure modification of Fe. When considered alongside the increased oxidation state revealed by XPS and sXAS, the reduced magnetic moment collectively signifies a substantial perturbation of the electronic configuration of Fe sites induced by the strong Lewis acidity of Ga^3+^.

**Fig. 4:**
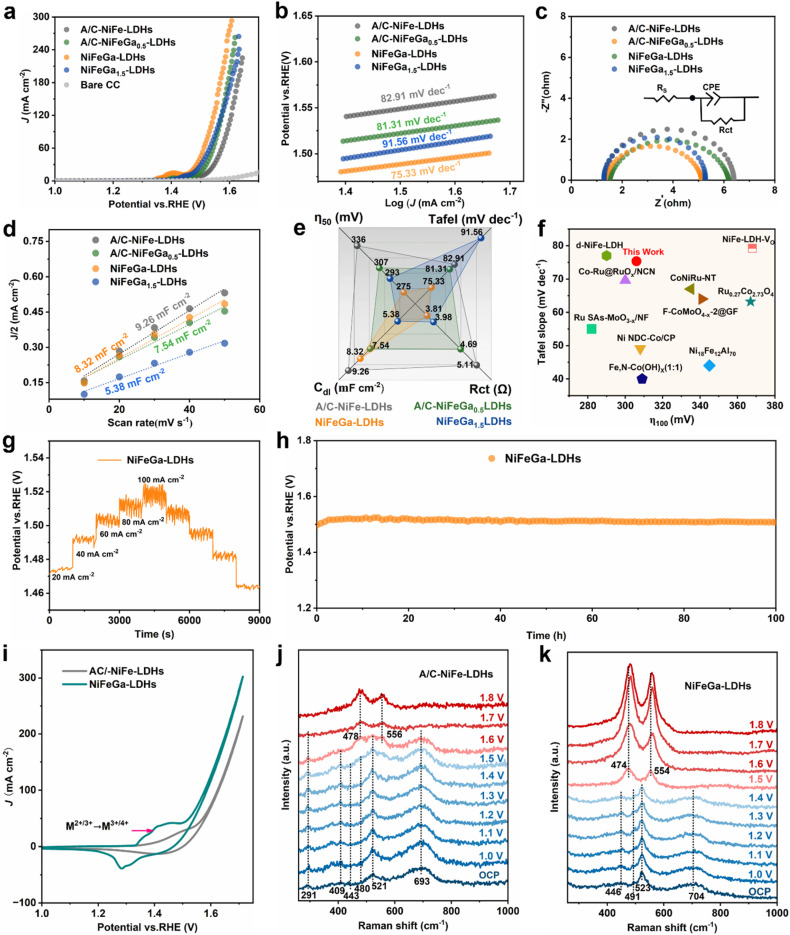
(a) Linear sweep voltammetry (LSV) curves of different samples in 1 M KOH at a scan rate of 5 mV s^−1^. (b) Tafel plots of different samples. (c) EIS Nyquist plots of different samples. (d) Electrochemical *C*_dl_ values of different samples. (e) Comparison of overpotentials *η*_50_, Tafel, *R*_ct_ and *C*_dl_ of different samples. (f) Comparison of the overpotentials (*η*_100_) and Tafel slopes between NiFeGa-LDHs and other recently reported catalysts. (g) Multi-current-step chronopotentiometry of NiFeGa-LDHs. (h) *V*–*t* curves of NiFeGa-LDHs at 50 mA cm^−2^. (i) Stable CVs after 20 potential cycles between 0.96 and 1.70 V *vs.* RHE. *Operando* Raman spectra of (j) A/C-NiFe-LDHs and (k) NiFeGa-LDHs.

The electrocatalytic activity of the synthesized catalysts for the oxygen evolution reaction was evaluated in a 1 M KOH electrolyte using a standard three-electrode configuration. As depicted in Fig. 4a, the NiFeGa-LDH catalyst exhibited markedly superior performance. It required an overpotential of merely 275 mV to deliver a current density of 50 mA cm^−2^, significantly lower than those of A/C-NiFe-LDHs (336 mV), A/C-NiFeGa_0_._5_-LDHs (307 mV), and NiFeGa_1_._5_-LDHs (293 mV). The enhanced OER activity is further corroborated by the Tafel slope analysis. NiFeGa-LDHs exhibit the smallest Tafel slope of 75.33 mV dec^−1^ (Fig. 4b), a significant reduction from 82.91 mV dec^−1^ for the undoped A/C-NiFe-LDHs, indicating more favorable reaction kinetics due to enhanced efficiency in the adsorption and desorption of intermediates. Charge transfer kinetics were probed by electrochemical impedance spectroscopy (EIS). The Nyquist plot in Fig. 4c reveals that NiFeGa-LDHs possess the smallest charge-transfer resistance (*R*_ct_ ≈3.81 Ω), signifying the most efficient charge transfer at the electrode–electrolyte interface. The corresponding equivalent circuit and fitting parameters are provided in the inset of Fig. 4c and Table S1. To evaluate the available active sites, the electrochemical active surface area (ECSA) was estimated from the double-layer capacitance (*C*_dl_) derived from cyclic voltammetry (Fig. S7). Interestingly, while the A/C-NiFe-LDH catalyst showed a slightly higher *C*_dl_ value (9.26 mF cm^−2^) than NiFeGa-LDHs (8.32 mF cm^−2^) (Fig. 4d), the latter demonstrated vastly superior activity. This disparity highlights that the exceptional performance of NiFeGa-LDHs is primarily attributable to their enhanced intrinsic activity rather than a larger surface area. Key performance parameters, including the overpotential at 50 mA cm^−2^ (*η*_50_), Tafel slope, charge transfer resistance (*R*_ct_), and double-layer capacitance (*C*_dl_), are comparatively summarized in Fig. 4e. Furthermore, the turnover frequency (TOF) and superior normalized ECSA of NiFeGa-LDHs quantitatively confirm their exceptional per-site efficiency and intrinsic catalytic activity (Fig. S8, S9 and Table S2).^39^ Remarkably, NiFeGa-LDHs demonstrated superior performance in 1 M KOH electrolyte compared to the recently reported NiFeCo-based catalysts (Fig. 4f and Table S3). The stability of the NiFeGa-LDH electrode was rigorously assessed through both short-term and long-term tests. During a multi-step chronopotentiometry test (Fig. 4g), the required potential at various current densities (20 to 100 mA cm^−2^) slightly decreased relative to the initial measurement, suggesting a benign activation process that optimizes the catalytic interface. Most importantly, a long-term chronopotentiometry test (Fig. 4h) demonstrated exceptional durability, with the catalyst exhibiting stable operation for 100 hours at a constant current density and exhibiting an overpotential increase of only ∼20 mV. To gain deeper insights into the structural origin of its durability, NiFeGa-LDHs were systematically characterized after the 100 hour stability test using XRD, SEM, TEM, and XPS analyses. XRD analysis (Fig. S10) reveals significantly weakened diffraction intensity compared to the initial sample, accompanied by the disappearance of sharp crystalline peaks. This substantial intensity reduction indicates a pronounced structural transition toward a more amorphous or disordered state following prolonged operation. SEM and TEM images (Fig. S11) confirm the retention of the interconnected nanosheet morphology with a porous architecture, demonstrating excellent robustness and maintained accessibility of active sites. XPS analysis further indicates a notable positive shift in the binding energy, with Ni 2p and Fe 2p spectra exhibiting shifts of 0.4 eV and 1.7 eV, respectively, consistent with an increase in the oxidation states of both metals and pointing to surface evolution into more active oxyhydroxide species during operation (Fig. S12). Collectively, these findings confirm that NiFeGa-LDHs preserve their overall structural coherence, nanoscale morphology, and chemically active surface state after prolonged OER testing, reinforcing their potential as a highly durable and efficient electrocatalyst. To understand the structural changes associated with the activation and decay of the LDH electrodes, we directly investigated their evolution under anodic OER conditions. The enhanced intrinsic activity of NiFeGa-LDHs is fundamentally linked to Ga-promoted electrochemical surface reconstruction, as unveiled by combined electrochemical and spectroscopic analysis. To elucidate this, stable cyclic voltammetry (CV) profiles of NiFeGa-LDHs and A/C-NiFe-LDHs were recorded after 20 potential cycles (0.96 to 1.70 V *vs.* RHE) (Fig. 4i). The NiFeGa-LDH catalyst exhibited the oxidation peak corresponding to the M^2+/3+^ to M^3+/4+^ transition at a lower onset potential compared to the undoped counterpart. Moreover, the integrated current for this redox process, which reflects the extent of active-site oxidation, was significantly larger for NiFeGa-LDHs. This indicates that Ga doping accelerates the electrochemical oxidation of Ni/Fe species, facilitating the formation of high-valent active phases. *Operando* Raman spectroscopy (Fig. 4j and k) provides direct structural evidence for this facilitated phase transformation. For A/C-NiFe-LDHs, characteristic peaks at ∼291 cm^−1^ (Fe(iii)–O) and ∼491 cm^−1^ (Ni(iii)–OOH bending) evolve with potential.^40^ A discernible feature at ∼409 cm^−1^, which may be tentatively assigned to a metal–oxygen vibration in the less-ordered structure, is also observed. The peaks at ∼443 and 521 cm^−1^, assignable to lattice vibrations of the pristine LDH layer, persist until 1.5 V. Above 1.6 V, they undergo a distinct blue shift to ∼478 and 556 cm^−1^, respectively, which are associated with the formation of γ-NiFeOOH^40,41^ (Fig. 4j). Notably, the peak at ∼693 cm^−1^, attributable to the Fe(iii)–OH vibration, also evolves and weakens with increasing potential.^42^ This sequence indicates a relatively sluggish and incomplete phase transformation, likely proceeding through an Fe(iii)–OH intermediate. It is noteworthy that, prior to this phase transformation, the Raman peaks of the pristine NiFeGa-LDHs (*e.g.*, 446, 491, 523, and 704 cm^−1^) are uniformly shifted to slightly higher wavenumbers compared to those of the undoped A/C-NiFe-LDHs (443, 480, 521, and 693 cm^−1^), suggesting an initial structural distinction. This subtle but consistent blue shift aligns with the sharper XRD peaks observed for NiFeGa-LDHs (Fig. 3a), potentially indicative of enhanced crystallinity or reduced structural disorder induced by Ga doping, which may pre-dispose the material to more facile reconstruction. Consequently, a drastic structural rearrangement then occurs as early as 1.5 V, with the initial peaks (446 and 523 cm^−1^) undergoing a blue shift to 474 and 554 cm^−1^ (Fig. 4k). Concomitantly, the Fe(iii)–OH peak (∼704 cm^−1^) diminishes and eventually vanishes as the potential increases. This signifies a Ga-promoted rapid conversion to γ-NiFe-OOH at lower potentials. Consistent with this accelerated kinetics, the γ-NiFeOOH phase not only emerges at a substantially lower potential in NiFeGa-LDHs (∼1.5 V *vs.* RHE) than in the undoped catalyst (∼1.6 V *vs.* RHE), but also exhibits markedly stronger characteristic Raman signal intensity at identical potentials, indicative of a more complete and rapid reconstruction. The dramatically lower onset potential and enhanced signal growth collectively demonstrate that Ga doping primarily accelerates the reconstruction kinetics, rather than fundamentally altering the identity of the final active phase. The subtle blue shift of the reconstructed phase's Raman peaks may indicate a minor modification of its local bonding environment, but the kinetic enhancement remains the predominant effect. These findings collectively demonstrate that the strong Lewis acidity of Ga^3+^ drives an earlier and more extensive reconstruction of the precatalyst into the active γ-NiFeOOH phase, which constitutes the key structural origin of its superior OER activity.

**Fig. 5:**
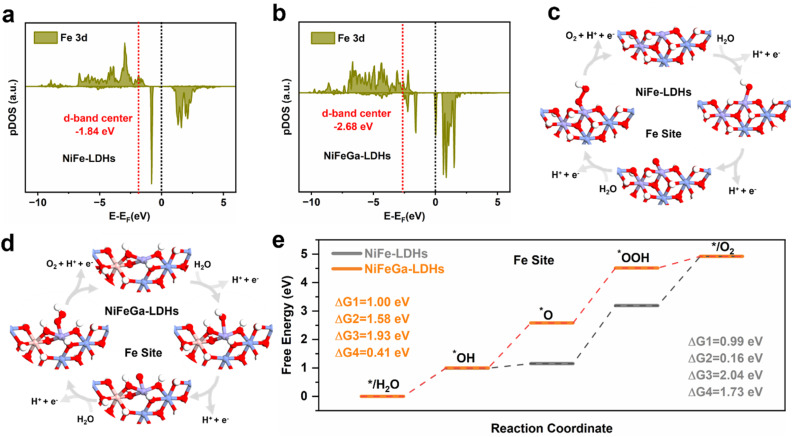
Projected density of states (pDOS) of active Fe atoms for (a) NiFe-LDHs and (b) NiFeGa-LDHs. Possible AEM pathway on the Fe site of (c) NiFe-LDHs and (d) NiFeGa-LDHs. (e) Gibbs free energy diagrams for NiFe-LDHs and NiFeGa-LDHs at *U* = 0 V on the Fe site.

To gain deeper insight into the origin of the enhanced activity, the electronic structure of the active sites was further probed through DFT calculations. Projected density of states (pDOS) analysis reveals distinct modulation trends near the Fermi level for the Fe sites in NiFeGa-LDHs, suggesting a facilitated electron transfer capability during catalysis (Fig. 5a and b). For the Fe sites, the d-band center (*ε*_d_) shifts downward from −1.84 eV to −2.68 eV. According to the d-band center theory, this shift signifies a moderated binding strength of the Fe sites with oxygen-containing intermediates, which is more favorable for the O–O bond formation step, thereby alleviating a key thermodynamic bottleneck in the OER.^21,43^ Concurrently, the Ni sites exhibit a pronounced upshift of the d-band center from −2.59 eV to −1.60 eV (Fig. S13a and b). This electronic restructuring, consistent with the Bader charge analysis indicating a pre-reduced Ni state, lowers the energy barrier for the oxidation of Ni^2+^ to higher valence states (Ni^3+^/Ni^4+^), thereby promoting its participation in the active phase. The thermodynamic feasibility of the OER was assessed by calculating the Gibbs free energy change (Δ*G*) for all elementary steps on the Ni and Fe active sites (Fig. 5c, d and S13c, d). The resulting free energy diagrams (Fig. 5e) indicate that Ga doping preferentially facilitates the formation of the critical *OOH intermediate. Specifically, on the Fe sites, the energy barrier for *OOH formation is calculated to be 0.70 eV for NiFeGa-LDHs, lower than the 0.81 eV for NiFe-LDHs. A similar promoting effect is observed on the Ni sites, with the corresponding barrier decreasing from 1.02 eV in NiFe-LDHs to 0.88 eV in NiFeGa-LDHs (Fig. S13e). This overall improvement can be rationalized by the strong Lewis acidity of Ga^3+^, which stabilizes the high oxidation state of Fe while concurrently activating the Ni centers, thereby optimizing the adsorption strength of key oxygenated intermediates and ultimately enhancing the OER catalytic activity.

**Fig. 6:**
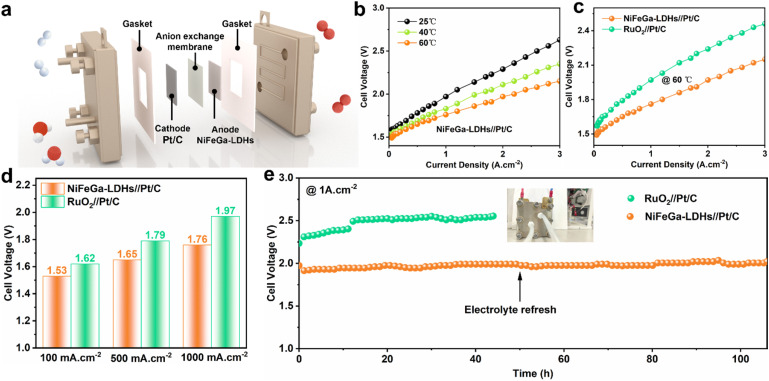
(a) Schematic illustration of the AEM reactor. (b) LSV performed at different cell temperatures in an AEM electrolyzer. (c) LSV curves of the AEM reactors using NiFeGa-LDHs and RuO_2_ as the anodic electrodes and the Pt/C catalyst as the cathodic electrode, respectively, at 60 °C. (d) Cell voltage comparison of NiFeGa-LDHs//Pt/C and commercial RuO_2_//Pt/C catalysts at various current densities (100, 500, and 1000 mA cm^−2^) in an AEM electrolyzer at 60 °C. (e) Stability tests of the AEM water electrolyzers employing NiFeGa-LDHs//Pt/C and commercial RuO_2_//Pt/C at 1 A cm^−2^.

To assess the practical application potential of NiFeGa-LDHs, an anion exchange membrane (AEM) electrolyzer was constructed under near-industrial conditions. The electrolyzer featured a sandwich configuration comprising current collectors, catalyst-coated electrodes, and anion membrane without hot pressing, as schematically illustrated in Fig. 6a. Commercial Pt/C was used as the cathodic catalyst, while the anodic performance of NiFeGa-LDHs was benchmarked against state-of-the-art RuO_2_ and Pt/C catalysts, all coated on commercially available 1 cm^2^ substrates. As anticipated, the overall electrolyzer performance improved with increasing operating temperature (Fig. 6b and S14). Remarkably, at 60 °C, the NiFeGa-LDH-based electrolyzer achieved voltages of only 1.53, 1.65, and 1.76 V at 100, 500, and 1000 mA cm^−2^, respectively (Fig. 6c and d), substantially outperforming the RuO_2_ and Pt/C benchmarks (1.97 V at 1 A cm^−2^). The operational stability was evaluated *via* chronopotentiometry at a constant current density of 1 A cm^−2^ for over 100 hours. As shown in Fig. 6e, the NiFeGa-LDH-based electrolyzer exhibited exceptional durability, with the cell voltage increasing only minimally from an initial 1.97 V to approximately 2.0 V after continuous operation. This outstanding stability, coupled with superior activity, presents a marked advantage over the benchmark RuO_2_ and Pt/C catalysts. These results collectively indicate that NiFeGa-LDHs are a highly promising anode material for efficient and stable hydrogen production in AEM electrolyzers.

## Conclusions

In summary, we have developed a targeted regulation strategy utilizing the strong electron-withdrawing capability of Ga^3+^ as a Lewis acid to modulate the electronic structure of Fe sites, thereby optimizing the intrinsic OER activity. The resulting NiFeGa-layered double hydroxide (NiFeGa-LDH) catalyst exhibits outstanding OER performance, achieving an overpotential of only 275 mV at 50 mA cm^−2^ in 1 M KOH. The AEM electrolyzer employing this catalyst delivers overall performance superior to that of the commercial RuO_2_//Pt-C benchmark, while exhibiting stable operation for over 100 hours at a high current density of 1 A cm^−2^. Through a combination of theoretical and experimental characterization, we reveal that Ga^3+^ acts as an “electron pump”, which effectively tunes and stabilizes the Fe active sites. This electronic modulation leads to electron depletion from Fe^3+^, resulting in an elevated valence state. Critically, *operando* Raman spectroscopic analysis combined with electrochemical measurements demonstrates that this optimized electronic environment drives an earlier and more extensive electrochemical reconstruction of the catalyst surface into the active γ-NiFeOOH phase under operating conditions. The Ga-induced electronic structure modification facilitates the electrochemical surface reconstruction, which collectively leads to an optimized adsorption of reaction intermediates on the reconstructed active sites. This thereby lowers the energy barrier for the potential-determining step and ultimately enhances the overall OER activity and durability. This work provides new insights into the design of highly active OER catalysts and offers a promising pathway toward their application in large-scale electrolysis systems.

## Author contributions

Y. Shuai designed and performed the experiments, data curation, calculations, formal analysis, writing the original draft, and writing – review and editing. S. C. Liu and G. B. Li discussed sample preparation, electrochemical data and DFT calculations. Y. Liu conceived the idea, conceptualization, methodology, supervision, funding acquisition, project administration, writing – review and editing. All authors have given approval to the final version of the manuscript.

## Conflicts of interest

There are no conflicts to declare.

## Supplementary Material

SC-017-D6SC00682E-s001

## Data Availability

The data supporting this article have been included as part of the supplementary information (SI). Supplementary information: experiments, characterization, calculations, additional spectroscopic data (XRD, SEM, TEM, XPS, GPA mappings, N_2_ adsorption, Hysteresis loops, sXAFS, ECSA, LSV, CV curves), and Tables S1–S3. See DOI: https://doi.org/10.1039/d6sc00682e.
