# Supplementary material for: Ga-induced electron pump optimization of Fe active sites in NiFe-LDHs for efficient alkaline water electrooxidation
Source: Chem Sci. 2026 Jun 18. Online ahead of print. doi: 10.1039/d6sc00682e (PMC13306958; doi:10.1039/d6sc00682e)
Supplement: SC-OLF-D6SC00682E-s001 [file SC-OLF-D6SC00682E-s001.pdf]

Supplementary Information (SI) for Chemical Science.
This journal is © The Royal Society of Chemistry 2026

Supporting Information

Ga-Induced Electron Pump Optimization of Fe active sites in NiFe-LDHs for Efficient Alkaline Water Electrooxidation

Yu Shuai [a], Shucheng Liu[a], GangBiao Li[a] and Yi Liu *[a]

a. School of Physical Sciences, Guizhou University, Guiyang 550025, China

*Corresponding author. E-mail address: yliu9@gzu.edu.cn

Pretreatment of Carbon Cloth (CC)

A piece of carbon cloth (CC) was first sonicated in 3 M HCl for at least 30 min, sequentially rinsed with deionized water and ethanol, and then dried in an oven at 60 °C. This pretreated CC was used as the substrate for the growth of all catalysts described below.

Synthesis of A/C-NiFe-LDHs

A homogeneous precursor solution was prepared by dissolving $Ni(NO_3)_2·6H_2O$ (1.2 mmol), $Fe(NO_3)_3·9H_2O$ (0.4 mmol), and urea (3.5 mmol) in 30 mL of deionized water under vigorous stirring. The resulting solution was transferred into a Teflon-lined stainless-steel autoclave, and a piece of the pretreated CC was immersed into it. The autoclave was sealed and heated at 120 °C for 8 h. After the autoclave was cooled to room temperature naturally, the resulting composite was collected, followed by centrifugation, ultrasonic washing with deionized water and ethanol several times, and finally drying at 80 °C for 10 h, yielding the final A/C-NiFe-LDHs.

Synthesis of Ga-doped NiFe-LDHs

Different from the A/C-NiFe-LDHs, the Ga-doped samples (denoted as A/C-$NiFeGa_{0.5}$-LDHs, NiFeGa-LDHs, and $NiFeGa_{1.5}$-LDHs) were synthesized using the same procedure but with varying molar ratios of the metal precursors: A/C-$NiFeGa_{0.5}$-LDHs: Ni^{2+} : Fe^{3+} : Ga^{3+} = 1.2 mmol : 0.3 mmol : 0.1 mmol; NiFeGa-LDHs: Ni^{2+} : Fe^{3+} : Ga^{3+} = 1.2 mmol : 0.2 mmol : 0.2 mmol; $NiFeGa_{1.5}$-LDHs: Ni^{2+} : Fe^{3+} : Ga^{3+} = 1.2 mmol : 0.1 mmol : 0.3 mmol.

Characterization methods

The morphology of the samples was measured by field emission scanning electron microscopy (SEM, Gemini300). High-resolution TEM (HRTEM, Tecnai G20) was employed to further demonstrate the lattice, and energy dispersive spectroscopy (EDS) elemental mapping was measured on this electron microscope. The phase structure, composition, and chemical morphology of the sample were characterized by X-ray diffraction (XRD, BRUCKER D8 ADVANCE X) with Cu Kα radiation and X-ray photoelectron spectroscopy (XPS, Escalab 250Xi, Thermo Fisher Scientific). Fe L-edge Soft X-ray absorption spectra (sXAS) were carried out at BL10B photoemission end-station of National Synchrotron Radiation Laboratory (NSRL, Hefei in China) in TEY mode. The spectra were processed and analyzed by the software Athena. For ZFC (Quantum Design-SQUID-VSM, MPMS-3) measurements, the samples were exposed to a temperature range of 2 to 450 K at a rate of 5 K/minutes, while an external field of 1000 Oe was applied. The specific surface of the samples were analyzed by BET tester (BSD-PM1, Bester Instrument Technology Co, Ltd, China). Regarding the TOF calculation, we utilized BET surface area measurements to determine the number of

active sites on the surface of both A/C-NiFe-LDHs and NiFeGa-LDHs. The N_2 adsorption-desorption isotherms of A/C-NiFe-LDHs and NiFeGa-LDHs, shown in Fig. S6, reveal their specific surface areas to be 192.91 m^2 g^{-1} and 177.76 m^2 g^{-1}, respectively. Using the following equation , we calculated the TOF for both catalysts at different current densities (50, 100, and 200 mA cm^{-2}):

$$TOF_{surface} = i \cdot N_A / F \cdot m \cdot S_g \cdot n \qquad (S1)$$

where i is the current density, N_A is Avogadro's number, F is Faraday's constant, m is the number of active sites per unit area, S_g is the specific surface area, and n is the number of electrons transferred per molecule. The calculated TOF for A/C-NiFe-LDHs and NiFeGa-LDHs is as shown in Table S2. In situ Raman spectra were acquired using inVia Qontor (Renishaw, Britain) instrument with a 532-nm laser. The in situ Raman spectroscopy was measured in a homemade cell with a flowing 1 M KOH electrolyte to remove oxygen bubbles and reduce their negative effect on collecting Raman signals. A three-electrode configuration was employed, with Hg/HgO, a Pt plate, and carbon cloth (CC) substrate with in-situ grown catalysts (0.5 mg cm^{-2}) serving as the reference, counter, and working electrodes, respectively. Other conditions were the same as electrochemical performance measurements.

Electrochemical measurements

Electrochemical measurements were performed with a CHI 760E station electrochemical analyzer in a standard three-electrode system: platinum plate as the counter electrode, Hg/HgO electrode as the reference electrode, and the as-prepared CC as the working electrode. The Hg/HgO electrode was calibrated against the reversible

hydrogen electrode (RHE), resulting in the following conversion equation:

$$E_{RHE} = E_{Hg/HgO} + 0.0591*pH + 0.098 \qquad (S2)$$

Prior to conducting the electrochemical measurements, all working electrodes were electrochemically stabilized by performing multiple cyclic voltammetry (CV) scans between 0.9 and 1.7 V (vs. RHE) at a scan rate of 50 mV s^{-1} until a stable response was achieved. The OER performance were tested in 1.0 M KOH using the linear sweep voltammetry (LSV) curves at a scan rate of 5 mV·s^{-1}. The Tafel slope (b) was calculated from $\eta = a + b \log(j)$ (j is the current density). The solution resistance (R_s) was obtained by impedance spectroscopy. This measurement was conducted at open circuit potential with the frequency set from 0.1 Hz to 100 kHz. From the diameter of the semicircle in the Nyquist plots, Rs was approximated. The potential was corrected by 100 % of Rs, according to the following equation:

$$E_{iRcorrected} = E - iRs. \qquad (S3)$$

The Tafel plots were derived from the LSV curves. The electrochemically active surface area (ECSA) can be estimated through the electrochemical double-layer capacitance (C_{dl}) collected from CV with different scan rates (10, 20, 30, 40, 50 mV·s^{-1}) in the potential range from 1.11 to 1.21 V (vs. RHE). Electrochemical impedance spectroscopy (EIS) was performed with an AC voltage with a 5 mV amplitude in the frequency range from 0.1 Hz to 100 kHz in open circuit potential with η = 300 mV. To assess the long-term water splitting stability of NiFeGa-LDHs, continuous chronopotentiometric V-t measurements were employed under a constant current density of 50 mA cm^{-2} for 100 hours. The ECSA value can be deduced through the

equation of ECSA = C_{dl}/C_s (C_s is taken as 0.04 mF·cm^{-2}).

Theoretical calculations

DFT Calculations were performed using the Vienna Ab-initio Simulation Package (VASP), employing the projector augmented wave (PAW) method and plane wave basis set.[1,2] The Perdew-Burke-Ernzerhof (PBE) functional within the Generalized S4 Gradient Approximation (GGA) was applied.[3,4] The Gibbs free energy (ΔG) for each reaction step was determined using the equation:

$$\Delta G = \Delta E + \Delta E_{ZPE} - T\Delta S \qquad (S4)$$

Here, ΔE is the reaction energy calculated under vacuum, while ΔE_{ZPE} and TΔS correspond to the zero-point energy correction from vibrational frequencies and the entropy change at 298.15 K, respectively. In our computational study of the oxygen evolution reaction (OER), we modeled the undercoordinated Ni/Fe-terminated (110) surface of NiFe-LDHs, which is widely adopted due to its relatively low surface energy. To eliminate spurious interactions between periodic images along the surface-normal direction, a vacuum layer of 15 Å was introduced in the c-axis. Parameters included a 400 eV cutoff energy and a 3 × 3 × 1 Monkhorst-Pack k-point grid. To consider the impact of the 3d electrons of the Ni and Fe atoms, the DFT + U method was utilized with effective U values of 3.4 eV and 3.3 eV, respectively. Convergence was set at 1×10^{-5} eV for energy and 0.01 eV/Å for forces. It should be noted that the DFT calculations were performed using an ordered lattice, as first-principles methods require periodic structures and modeling a fully amorphous quaternary system is highly challenging. The model is constructed to elucidate how the Ga^{3+} dopant modulates the

local electronic structure of the Fe active sites and thereby tunes the adsorption energetics of key reaction intermediates, rather than to achieve an exact structural match of the amorphous bulk catalyst. Although the experimental catalyst is disordered at long range, its short range metal-oxygen coordination closely matches the crystalline model. The calculated trends in electronic structure and adsorption energies agree with XPS, surface-sensitive soft X-ray absorption spectroscopy (XAS), and catalytic activity measurements, supporting the relevance of the model.

Assembly of anion exchange membrane (AEM) water electrolysis

The anion exchange membrane (AEM) water electrolyze system consists of four components: power supply, temperature control system, flow control system, and anion exchange membrane water solution. The electrolyser consists of two corrosion-resistant TA1 grade plates with SUS 316 end plates and a curved flow field machined into the bipolar plates. The anode and cathode electrodes are separated by an anion-exchange membrane to prevent gas crossings and are pressed against the sides of the electrolyze to form a membrane electrode assembly (MEA). The MEA was made of an anion exchange membrane (A40-HCO3, 40 μm thick) with cathode (Pt/C on carbon paper) and anode (NiFeGa-LDHs on nickel foam) catalysts, both with an area of 1 cm^2 and a loading of ~1 mg cm^{-2}, located on both sides of the membrane. The electrolytic cell was equipped with a temperature control system (25-60°C) and used 1 M KOH as the electrolyte.

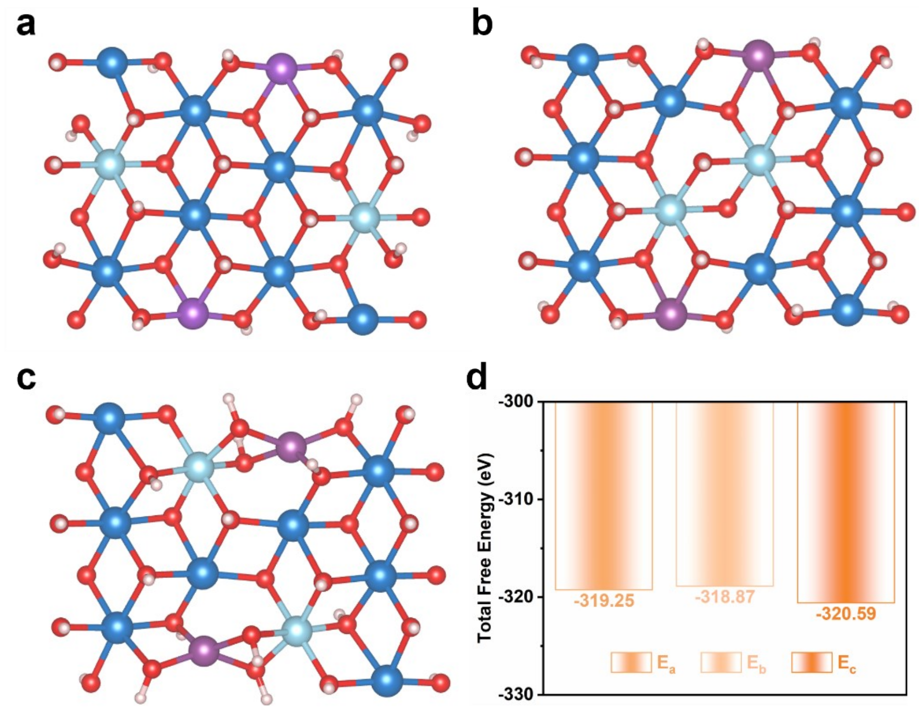

Fig. S1 (a-c) Different periodic substitution configurations of NiFeGa-LDHs and (d) and their formation energy.

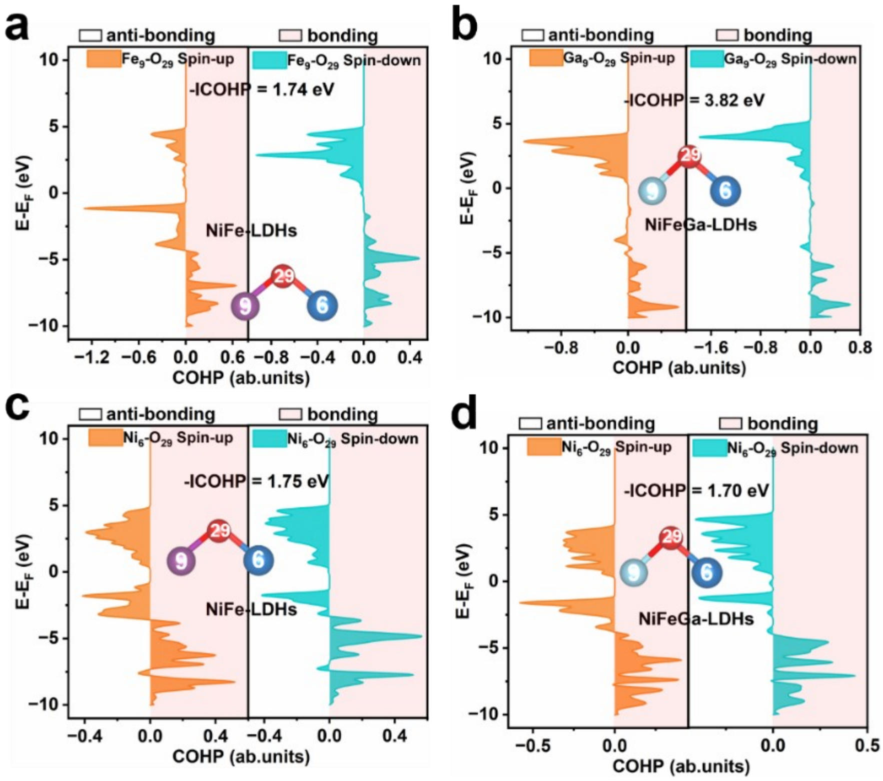

Fig. S2 Comparative crystal orbital Hamilton population (COHP) analysis near the active Ni site in (a,c) NiFe-LDHs and (b,d)NiFeGa-LDHs.

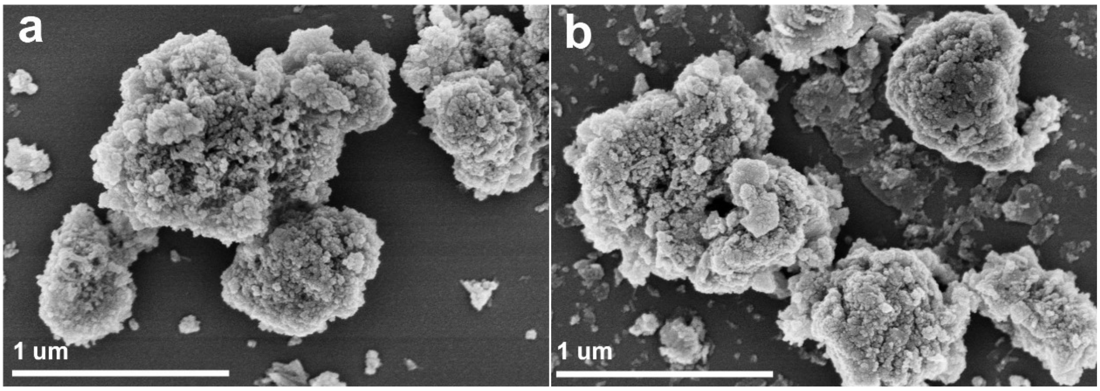

Fig. S3 SEM images of (a) A/C-NiFe-LDHs and (b) NiFeGa-LDHs.

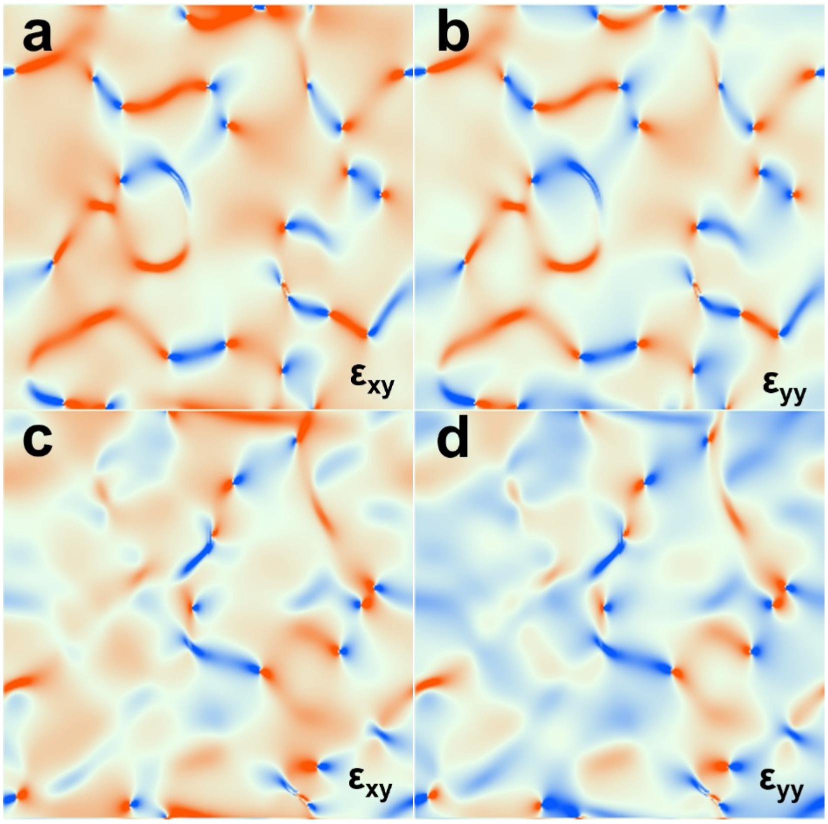

Fig. S4 ε_{xy}, ε_{yy} geometric phase analysis (GPA) mappings between (a,b) A/C-NiFe-LDHs and (c,d) NiFeGa-LDHs.

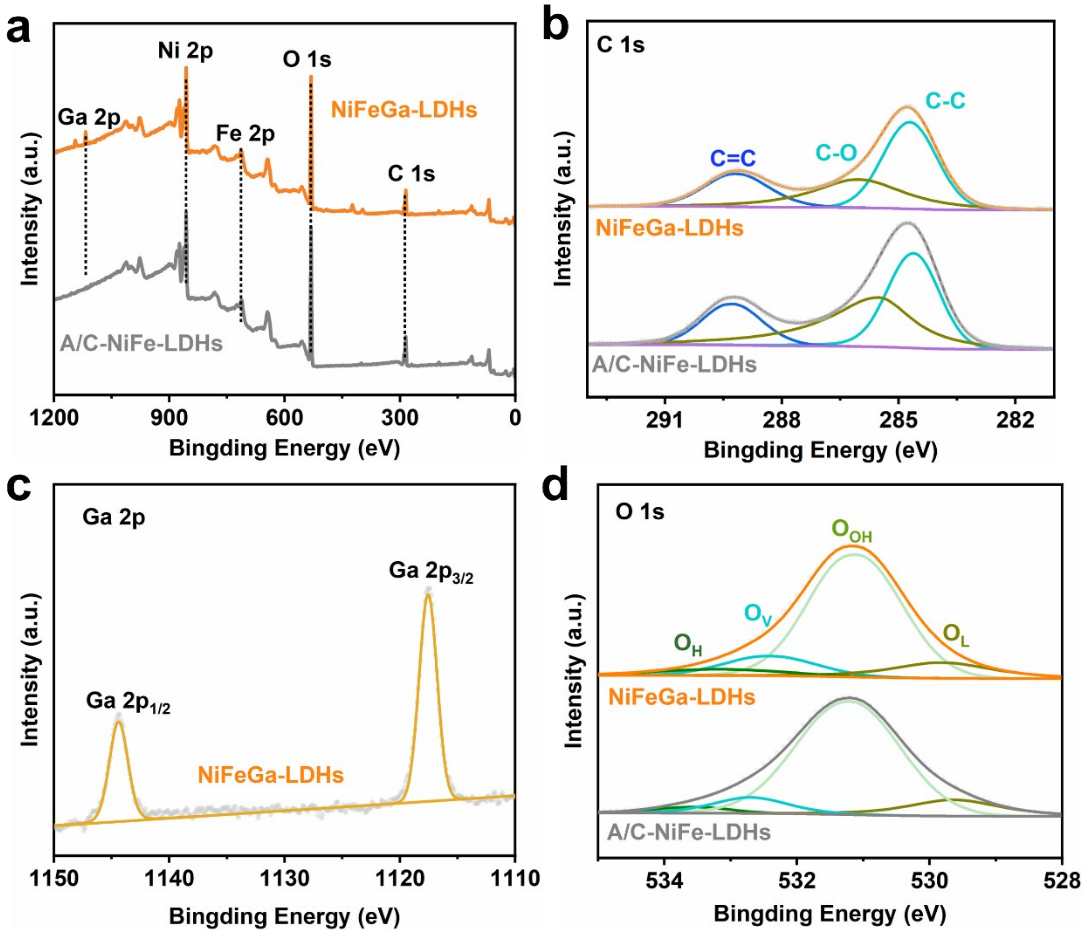

Fig. S5 (a) XPS survey spectrum. (b) XPS scans of C 1s of A/C-NiFe-LDHs and NiFeGa-LDHs. (c) XPS scans of Ga 2p of NiFeGa-LDHs. (d) XPS scans of O 1s of A/C-NiFe-LDHs and NiFeGa-LDHs.

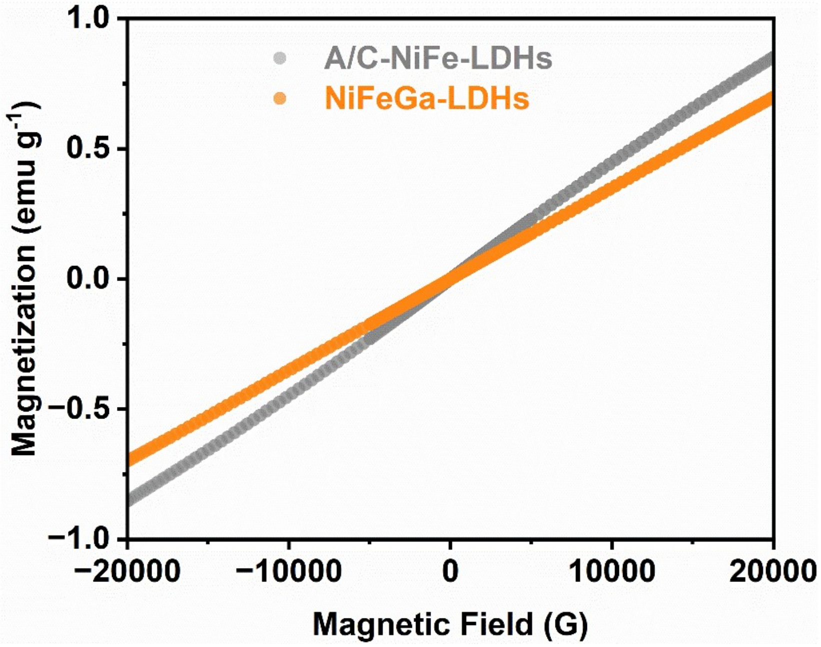

Fig. S6 Hysteresis loops of A/C-NiFe-LDHs and NiFeGa-LDHs.

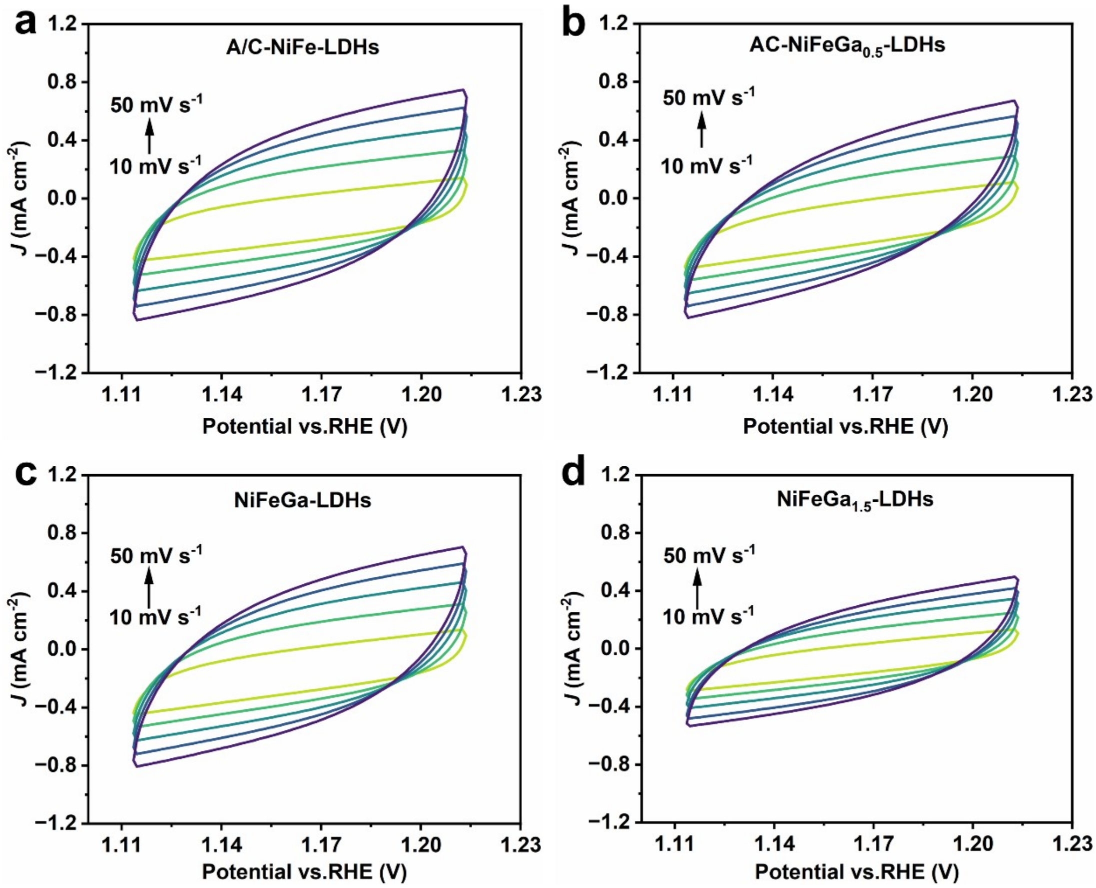

Fig. S7 CV curves of (a) A/C-NiFe-LDHs, (b) A/C-NiFe$Ga_{0.5}$-LDHs, (c) NiFeGa-LDHs and (d) NiFe$Ga_{1.5}$-LDHs.

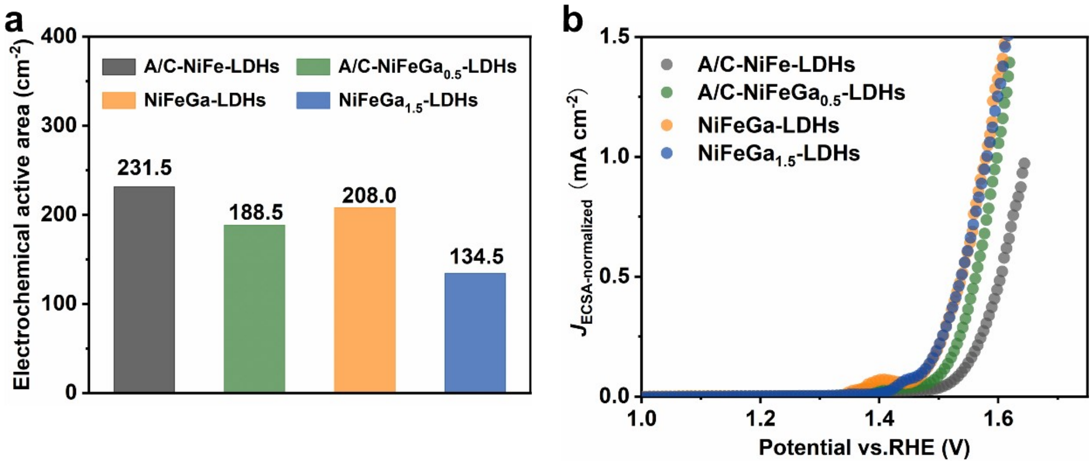

Fig. S8 (a) ECSA values of of different samples. (b) OER polarization curves after normalized by ECSA.

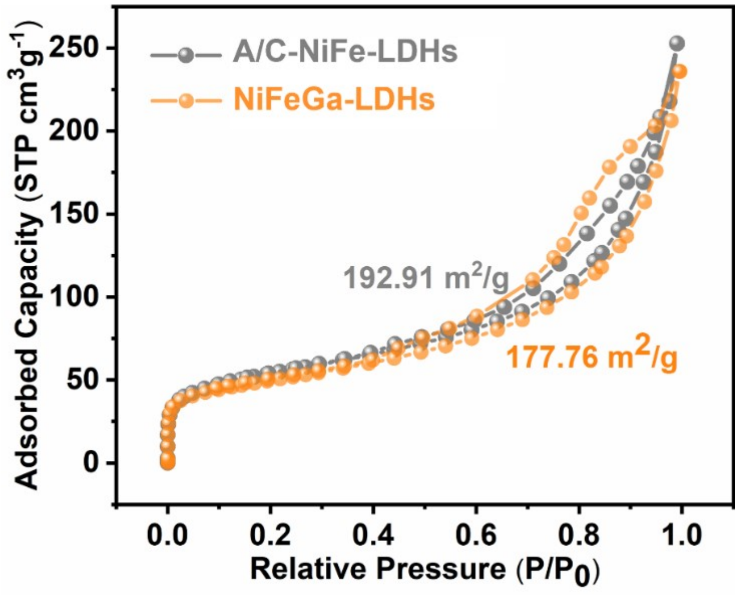

Fig. S9 N_2 adsorption-desorption isotherms of A/C-NiFe-LDHs and NiFeGa-LDHs.

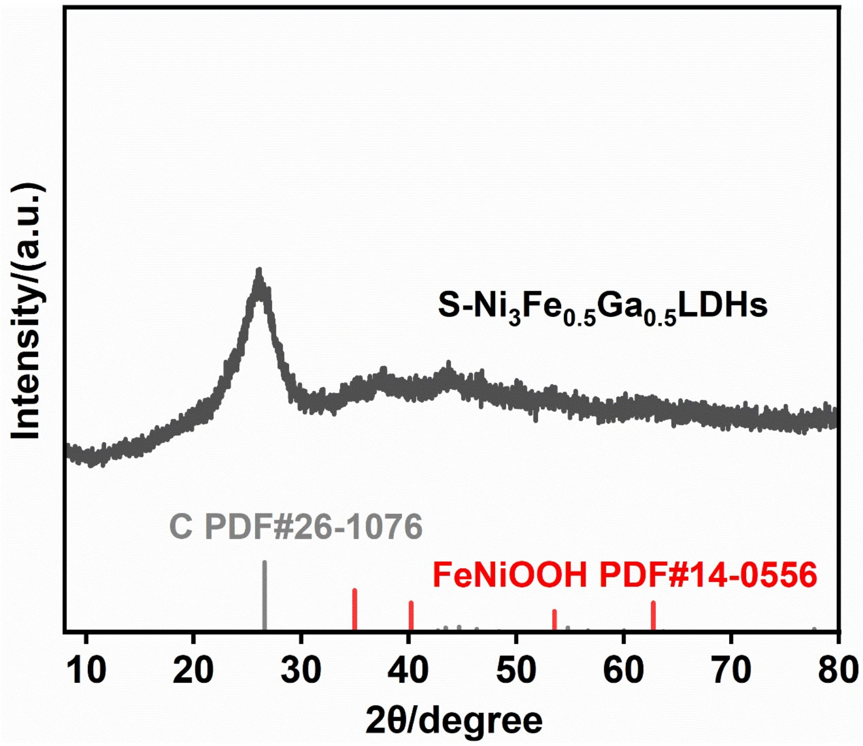

Fig. S10 XRD of NiFeGa-LDHs after durability measurement.

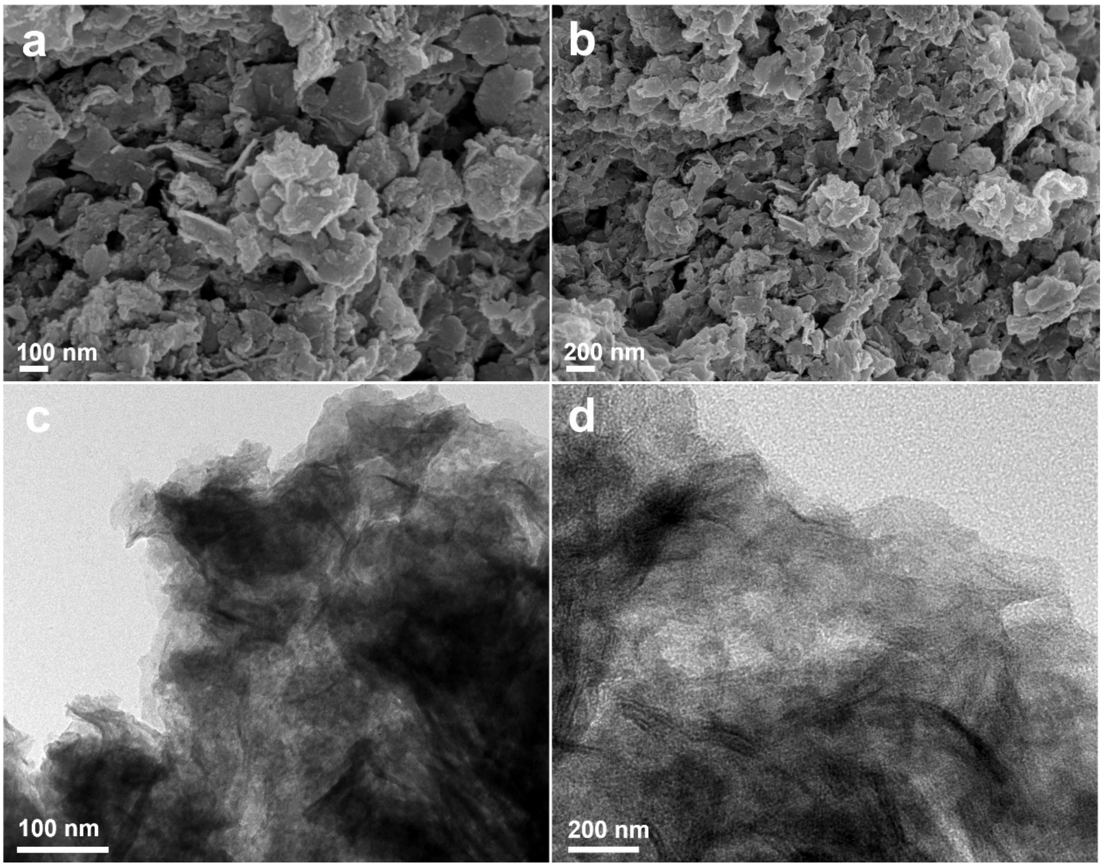

Fig. S11 (a-b) SEM and (c-d) TEM images of NiFeGa-LDHs after durability measurement.

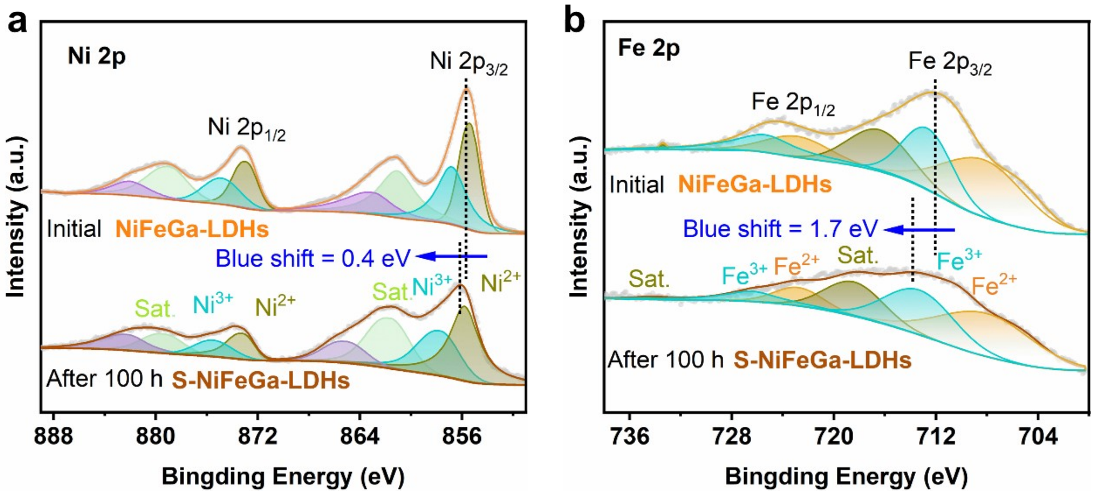

Fig. S12 (a) Ni 2p and (b) Fe 2p XPS spectra of NiFeGa-LDHs before and after durability measurement.

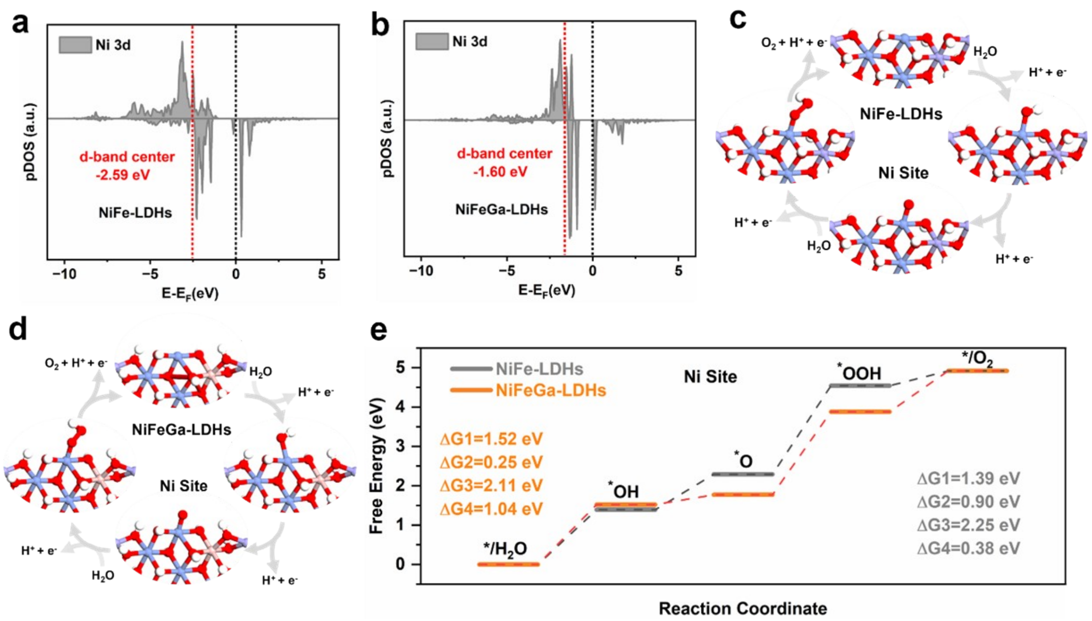

Fig. S13 Project density of states (pDOS) of active Ni atoms for (a) NiFe-LDHs and (b) NiFeGa-LDHs. Possible AEM pathway on the Ni site of (c) NiFe-LDHs and (d) NiFeGa-LDHs. (e) Gibbs free energy diagram on NiFe-LDHs and NiFeGa-LDHs at U = 0 V of Ni site.

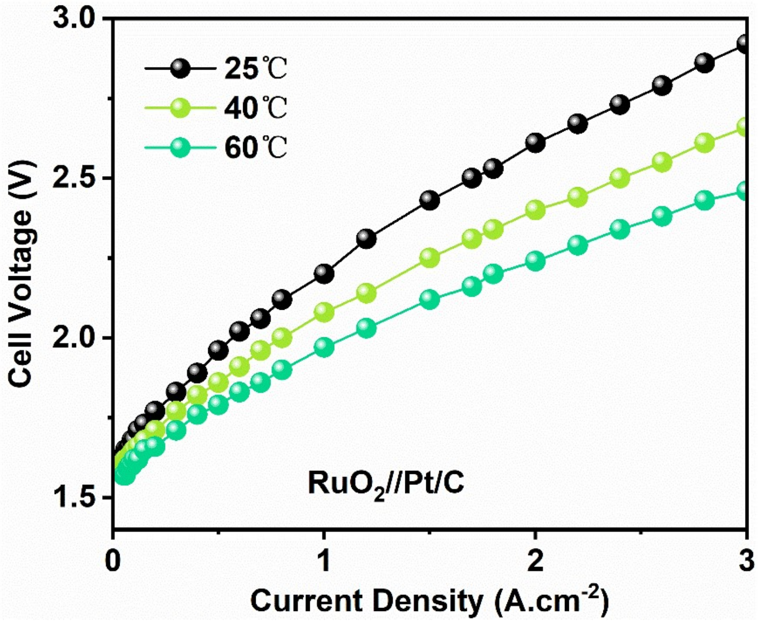

Fig. S14 LSVs operated at different cell temperatures in an AEM electrolyzer using RuO_2 and Pt/C catalysts as the anodic and cathodic electrodes, respectively.

Table S1. The values of series resistance (R_s) and charge transfer resistance (R_{ct}) for samples in 1M KOH.

Samples	R_S (Ω)	R_{ct} (Ω)
A/C-NiFe-LDHs	1.28	5.11
A/C-NiFeGa$_{0.5}$-LDHs	1.50	4.69
NiFeGa-LDHs	1.32	3.81
NiFeGa$_{1.5}$-LDHs	1.29	3.98

Table S2. The TOF of NiFe-LDHs and NiFeGa-LDHs at different current densitys

Samples	50 mA/cm^{-2}	100 mA/cm^{-2}	200 mA/cm^{-2}
A/C-NiFe-LDHs	1.69 s^{-1}	3.37 s^{-1}	6.73 s^{-1}
NiFeGa-LDHs	1.83 s^{-1}	3.65 s^{-1}	7.30 s^{-1}

Table S3. Comparison of overpotentials at 100 mA cm^{-2} current densities and the

corresponding Tafel slope of various catalysts in 1.0 M KOH electrolyte.

Catalysts	η@100 mA cm^{-2} (mV)	Tafel Slope (mV dec^{-1})	Reference
Ru SA_s-MoO_{3-x}/NF	282	55.0	***Adv. Sci.*** 2023, 10, 2300342.[5]
d-NiFe-LDH	290	77.0	***Angew. Chem.-Int. Edit.*** 2021, 60, 26829.[6]
Co-Ru@RuO_x/NCN	300	69.6	***J. Energy Chem.*** 2023, 87, 286-294.[7]
NiFeGa-LDHs	306	75.3	**This work**
Ni NDC-Co/CP	308	49.1	***Inorg. Chem.*** 2024, 63, 7045−7052.[8]
Fe,N-$Co(OH)_x$(1:1)	309	40	***EES Catal.,*** 2025, 3, 407.[9]
CoNiRu-NT	335	67.0	***Adv. Mater.*** 2022, *34*, 2107488.[10]
F-$CoMoO_{4-x}$-2@GF	341	64	***Appl. Catal. B*** 2022, 303, 120871.[11]
$Ni_{18}Fe_{12}Al_{70}$	345	44	***Angew. Chem. Int.*** 2023, 62, e202300800.[12]
$Ru_{0.27}Co_{2.73}O_4$	367	63.2	***Small.*** 2024, 20, 2310372.[13]
NiFe-LDH-V_O	368	79.2	***Chem. Eng. J.*** 2022, 446, 137226.[14]

References

1. G. K. A and J. F. b, Efficiency of ab-initio total energy calculations for metals and semiconductors using a plane-wave basis set - ScienceDirect, *Computational Materials Science*, 1996, **6**, 15–50.
2. P. Blochl, E. Blöchl and P. E. Blöchl, Projected augmented-wave method, *Phys. Rev. B.*, 1994, **50**, 17953.
3. John, P., Perdew, Kieron, Burke, Matthias, Ernzerhof and Erratum, Generalized Gradient Approximation Made Simple, *Physical Review Letters*, 1996. **77**, 3865-3868.
4. G. Kresse and D. Joubert, From ultrasoft pseudopotentials to the projector augmented-wave method, *Physical Review B*, 1999, **59**, 1758–1775.
5. D. Feng, P. Wang, R. Qin, W. Shi, L. Gong, J. Zhu, Q. Ma, L. Chen, J. Yu, S. Liu and S. Mu, Flower-Like Amorphous MoO_{3-x} Stabilized Ru Single Atoms for Efficient Overall Water/Seawater Splitting,

Advanced Science, 2023, **10**, 2300342.

6. Y. Wu, J. Yang, T. Tu, W. Li, P. Zhang, Y. Zhou, J. Li, J. Li and S. Sun, Evolution of Cationic Vacancy Defects: A Motif for Surface Restructuration of OER Precatalyst, *Angew. Chem.-Int. Edit.*, 2021, **60**, 26829–26836.
7. H. Wang, P. Yang, X. Sun, W. Xiao, X. Wang, M. Tian, G. Xu, Z. Li, Y. Zhang, F. Liu, L. Wang and Z. Wu, Co-Ru alloy nanoparticles decorated onto two-dimensional nitrogen doped carbon nanosheets towards hydrogen/oxygen evolution reaction and oxygen reduction reaction, *J. Energy Chem.*, 2023, **87**, 286–294.
8. H. Yin, X. Liu, L. Wang, T. Isimjan, D. Cai and X. Yang, Real Active Site Identification of Co/Co_3O_4 Anchoring Ni-MOF Nanosheets with Fast OER Kinetics for Overall Water Splitting, *Inorg. Chem.*, 2024, **63**, 7045–7052.
9. Q. Yang, Y. Li, Y. Wu, Y. Li, C. Yang, L. Ban, Y. Zhao, B. Dai, G. Wang, Y. Li, J. Zhang, Z. Wang, H. Pang and F. Yu, A plasma-triggered N–Co–O–Fe motif in $Co(OH)_2$ for efficient electrocatalytic oxygen evolution, *EES Catalysis*, 2025, **3**, 407–419.
10. Y. Wang, S. Wang, Z. Ma, L. Yan, X. Zhao, Y. Xue, J. Huo, X. Yuan, S. Li and Q. Zhai, Competitive Coordination-Oriented Monodispersed Ruthenium Sites in Conductive MOF/LDH Hetero-Nanotree Catalysts for Efficient Overall Water Splitting in Alkaline Media, *Adv. Mater.*, 2022, **34**, 202300342.
11. W. Xie, J. Huang, L. Huang, S. Geng, S. Song, P. Tsiakaras and Y. Wang, Novel fluorine-doped cobalt molybdate nanosheets with enriched oxygen-vacancies for improved oxygen evolution reaction activity, *Appl. Catal. B-Environ. Energy*, 2022, **303**, 120871.
12. X. Liu, H. Lu, S. Zhu, Z. Cui, Z. Li, S. Wu, W. Xu, Y. Liang, G. Long and H. Jiang, Alloying-Triggered Phase Engineering of NiFe System via Laser-Assisted Al Incorporation for Full Water Splitting, *Angew. Chem.-Int. Edit.*, 2023, **62**, 202300800.
13. G. Wang, G. Zhang and X. Chen, Ru Single Atoms Integrated into Cobalt Oxide Spinel Structure with Interstitial Carbon for Enhanced Electrocatalytic Water Oxidation, *Small*, 2024, **20**, 202310372.
14. H. Su, J. Jiang, N. Li, Y. Gao and L. Ge, NiCu alloys anchored defect-rich NiFe layered double-hydroxides as efficient electrocatalysts for overall water splitting, *Chem. Eng. J.*, 2022, **446**, 137226.
